# Maternal Prepregnancy Weight and Pregnancy Outcomes in Saudi Women: Subgroup Analysis from Riyadh Mother and Baby Cohort Study (RAHMA)

**DOI:** 10.1155/2021/6655942

**Published:** 2021-04-01

**Authors:** Hayfaa Wahabi, Samia Esmaeil, Amel Fayed

**Affiliations:** ^1^Chair for Evidence-Based Health Care and Knowledge Translation, College of Medicine, King Saud University, Riyadh, Saudi Arabia; ^2^Department of Community and Family Medicine, King Saud University, Riyadh, Saudi Arabia; ^3^College of Medicine, Princess Nourah Bint Abdulrahman University, Riyadh, Saudi Arabia; ^4^Department of Biostatistics, High Institute of Public Health, Alexandria University, Alexandria, Egypt

## Abstract

The objectives of this study were to estimate the prevalence of prepregnancy overweight/obesity and underweight among Saudi mothers and to determine the adverse pregnancy outcomes associated with them. *Methods*. This is a subgroup analysis from a Riyadh mother and baby cohort study. Participants were divided into four groups according to prepregnancy BMI. Participants with normal BMI were the reference group. Groups were compared in relation to pregnancy-related obstetric, as well as fetal and neonatal complications. A regression model was used to control for covariates, and adjusted odds ratios (AOR) with 95% Confidence Intervals (95% CI) were calculated. *Results*. A total of 7,029 women were included, 29.7% had normal BMI, 33.3% were overweight, 34.8% were obese, and 2.2% were underweight. Obesity was associated with increased odds of gestational diabetes (AOR 2.07, 95% CI 1.73-2.47), hypertensive events in pregnancy (AOR 2.33, 95% CI 1.19-3.91), induction of labour (IOL) (AOR 1.40, 95% CI, 1.19-1.65), failed IOL (AOR 2.13, 95% CI 1.40-3.25), and delivery by emergency caesarean section (CS) (AOR 1.67, 95% CI 1.39-2.01). Infants of obese women had increased odds of macrosomia (AOR 3.73, 95% CI 2.33-5.98). Overweight women had increased odds of CS delivery (AOR 1.25, 95% CI 1.03-1.5) and failed IOL (AOR 1.69, 95% CI 1.09-2.60). Underweight women had increased odds of delivering a low birth weight (LBW) infant (AOR 2.49, 95% CI, 1.58-3.92). *Conclusion*. The prevalence of prepregnancy overweight and obesity is very high in Saudi Arabia. Prepregnancy obesity is associated with GDM and hypertensive events inpregnancy, IOL, failed IOL, and CS delivery. Infants of obese mothers were at higher risk of macrosomia, while underweight women were at increased risk of delivering LBW infants.

## 1. Introduction

Maternal prepregnancy weight is known to influence maternal and perinatal outcomes [1, 2] as well as the child and adult future health [3, 4].

Obesity is a global health problem. In 2016, nearly two billion adults were overweight, of whom 650 million were obese, worldwide [5]. The pandemic of obesity is not limited to high-income countries, as an increasing number of low and middle-income countries (LMIC) are reporting high prevalence of overweight and obesity [5]. In Saudi Arabia, the prevalence of adult obesity has increased from 35% to 50% in 15 years [6] [7], with a noticeable increase of the prevalence of overweight and obesity among pregnant women, from 52% to 68% [8] [9] in a decade.

Maternal obesity is associated with many adverse pregnancy outcomes. A recent systematic review of cohort studies from North America and Europe estimated that 23.9% of pregnancy complications were caused by maternal overweight/obesity [10]. Many studies confirmed the detrimental effects of maternal overweight, irrespective of other morbidities such as gestational diabetes (GDM), on pregnancy outcomes [11, 12]. Such adverse outcomes include increased rates of delivery by caesarean section (CS), hypertensive disorders of pregnancy, GDM, large for gestational age infants, and stillbirths (SB) [13, 14]. Furthermore, infants born to overweight mothers are at increased risk of childhood and adulthood obesity, cardiac, and metabolic diseases in addition to infant mortality [3, 15].

Although maternal underweight is not as prevalent as maternal overweight, it is yet associated with preterm birth (PTB) and low birth weight (LBW) [16] and both are recognized proximal events for under-five mortality, especially in LMIC [17].

The prevalence of underweight is relatively low in Saudi Arabia and varies between regions, from 2.2% in Riyadh, the capital city, to 8.5% in the eastern province [9] [8].

Quantifying the prevalence of obesity, underweight, and the associated pregnancy outcomes in any community is important for identifying pregnancies at higher risk of adverse outcomes [18]. Such knowledge will facilitate the strategic planning of health services to target the population at higher risk with the needed healthcare [19] and preventive measures [20].

Only a few studies have addressed the effect of maternal weight on pregnancy outcomes in Saudi Arabia [11, 21], some of which included a relatively small number of participants and were published more than a decade ago [8].

The objectives of this subcohort analysis from the Riyadh Mother and Baby Multicenter Cohort study (RAHMA) are the following:

1. To estimate the prevalence of overweight/obesity and underweight in a large cohort of Saudi mothers
2. To determine the adverse maternal and perinatal outcomes associated with overweight/obesity and underweight mothers compared to normal weight ones

## 2. Methods

RAHMA is a large multicenter cohort study with the main objective of investigating risk factors of adverse pregnancy outcomes in Saudi Arabia. The study recruited more than 14,500 Saudi pregnant women and their infants in three hospitals in Riyadh.

Participants of the RAHMA study completed a self-administered questionnaire which provided information on the socioeconomic status and antenatal history. Additionally, all obstetric and laboratory data of the women's medical records were added to the database of the study. Further details of the RAHMA study design can be found in Wahabi et al. [9].

For the current report, all Saudi women who fulfilled the following criteria were included:

1. Gestational age of 24 weeks or more at the time of delivery, calculated according to the last menstrual period and/or the early fetal ultrasound
2. Singleton pregnancy
3. Documented prepregnancy weight and first trimester height

The exclusion criteria were women with medical conditions that may affect the weight such as hypo- or hyperthyroidism, diagnosed eating disorders, organ transplantation, renal disease, cardiac disease, sickle cell disease, and multiple pregnancies.

We excluded 7339 participants due to missing data on prepregnancy weight. The comparison between the characteristics of the included and the excluded participants is available in supplementary 1 file.

Participants were divided, according to the WHO classification of body mass index (BMI), into four groups: underweight, normal BMI, overweight, and obese women. The groups were compared in relation to sociodemographic characteristics, such as education level, age, passive exposure to tobacco smoke, and working status. In addition, medical and obstetric conditions including chronic diseases such as diabetes mellitus (DM), preexisting hypertension, parity, occurrence of pregnancy complications, and gestational age at delivery were also recorded. The effects of prepregnancy BMI were investigated with respect to the following maternal and neonatal outcomes: maternal admission to the intensive care unit (ICU), gestational hypertension, development of GDM, induction of labour (IOL), delivery by CS, APGAR score less than 7 in the fifth minute of life, PTB, LBW, intrauterine growth restriction (IUGR), macrosomia, admission to the neonatal intensive care unit (NICU), and stillbirths.

### 2.1. Definitions

The following definitions were considered for this study:

(1) Maternal prepregnancy BMI was classified according to the WHO weight classification [22]:
  - Underweight (<18.5 kg/m^2^)
  - Normal (18.5-24.9 kg/m^2^)
  - Overweight (25.0-29.9 kg/m^2^)
  - Obese (≥30 kg/m^2^)
(2) Gestational diabetes mellitus was diagnosed based on the WHO criteria at any time in pregnancy if one or more of the following criteria were met [23]:
  - Fasting plasma glucose of 5.1–6.9 mmol/L (92–125 mg/dL)
  - One-hour plasma glucose ≥ 10.0 mmol/L (180 mg/dL) following a 75 g oral glucose load
  - Two-hour plasma glucose of 8.5–11.0 mmol/L (153–199 mg/dl) following a 75 g oral glucose load
(3) Gestational hypertension is defined as a new episode of elevated blood pressure (≥140 mmHg systolic or ≥90 mmHg diastolic on at least two occasions, 6 h apart) after 20 weeks of gestation in a previously normotensive woman, preeclampsia is defined as the new onset of elevated blood pressure after 20 weeks of gestation in a previously normotensive woman (≥140 mmHg systolic or ≥90 mmHg diastolic on at least two occasions 6h apart) in addition to proteinuria of at least 1+ on a urine dipstick or ≥300 mg in a 24 h urine collection, and superimposed preeclampsia is defined as new episode of preeclampsia after 20 weeks of pregnancy in a previously hypertensive woman [24]
(4) Macrosomia is defined as a birth weight of ≥4.0 kg
(5) LBW is defined as a birth weight < 2.5 kg
(6) Intrauterine growth restriction was considered if the clinical diagnosis was reported as such in the medical records based on fetal biometry and amniotic fluid volume less than expected for gestational age detected by antenatal ultrasound scan
(7) Postdate pregnancy is defined as a pregnancy that continues past 41 completed weeks of gestation [25]
(8) PTB is defined as a birth that takes place before 37 weeks of gestation. It is further subclassified as PTB (34–36 weeks) and (<34 weeks of gestation) [26]
(9) Passive exposure to tobacco smoke (PETS) is considered significant if the pregnant woman is living with a smoker (husband or other relative), who smokes at home, or if she works in an office with a smoker who smokes in the same office

### 2.2. Statistical Analysis

Data were analysed using the Statistical Package for the Social Sciences (SPSS), version 20 (SPSS Inc., Chicago, IL, USA). An Analysis of Variance (ANOVA) test was used to compare quantitative variables (after testing them for normality), and the chi-squire test was used to test associations of categorical variables and the four BMI groups. Multivariate logistic regression models were developed to adjust for known clinical confounders including maternal age, gestational age, and parity for all the outcomes excepting for PTB, which was adjusted for maternal age, parity, preexisting diabetes, gestational hypertension, and GDM. Normal weight women were considered the reference group. Age, parity, and gestational weight were considered in the models as continuous variables. Crude and adjusted odds ratios (AOR) with 95% Confidence Intervals (95% CI) were reported. *P* values of less than 0.05 were considered significant.

### 2.3. Ethical Considerations

The ethical approval for the main cohort study (RAHMA) was provided by the following institutions: King Abdullah International Medical Research Centre, approval letter 11/062; King Fahad Medical City Research Centre, approval letter 013–017; and King Saud University, approval letter 13–985. The study was conducted according to the principles of Helsinki Declaration.

## 3. Results

A total of 7,029 women were included in this study, of whom 2,087 (29.7%) had normal prepregnancy weight, 2,338 (33.3%) were overweight, and 2,447 (34.8%) were obese, while only 157 (2.2%) were underweight. The obese and overweight groups were older, of higher parity and less educated compared to normal weight women (Table 1). In addition, a small proportion of obese and overweight women had paid jobs (Table 1). The prevalence of preexisting diabetes and hypertension was significantly higher in overweight and obese women, with a noticeable increase in the frequency with the increment of BMI (Table 1).

The underweight women were the youngest group of the study population (mean of 26 years ±4.5 of SD), had the lowest parity and were the least passively exposed to tobacco smoke. However, similar to the other groups, they were mostly housewives (Table 1). In addition, underweight women did not suffer from chronic diseases (Table 1). PETS was not significantly different among all BMI groups.

**Figure 1:**
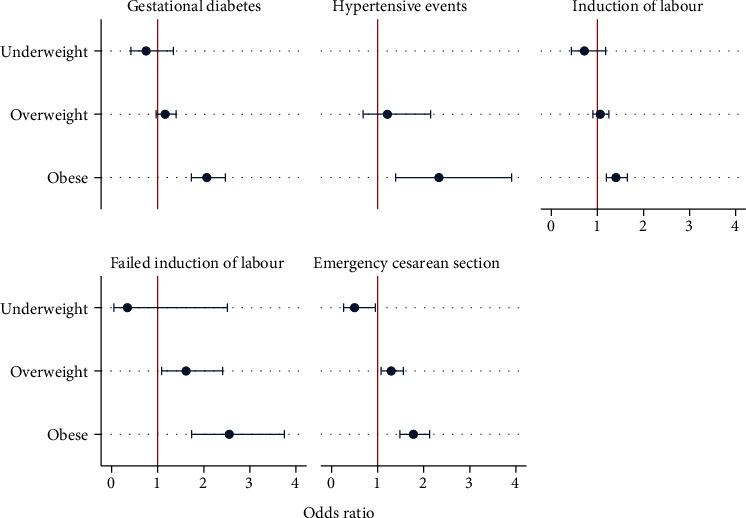
Adjusted association between prepregnancy body mass index and maternal outcomes.

**Figure 2:**
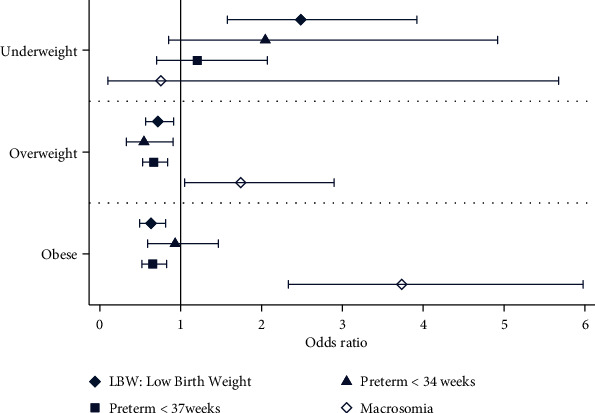
Adjusted association between prepregnancy body mass index and neonatal outcomes.

Compared to normal weight women, for obese women, the odds of developing GDM were increased by twofold (AOR 2.07, 95% CI 1.73-2.47), the odds of hypertensive events (gestational hypertention, pre-eclampsia and superimposed pre-eclampsia) were increased by almost two and half fold (AOR 2.33, 95% CI 1.19-3.91), the odds for IOL were increased by almost one and half fold (AOR 1.40, 95% CI, 1.19-1.65), and those for failed IOL were increased by more than twofold (AOR 2.13, 95% CI 1.40-3.25) (Tables 2 and 3). Furthermore, obese women were at increased risk of emergency CS by almost twofold (AOR 1.67, 95% CI 1.39-2.01), compared to normal weight women, (Tables 2 and 3, Figure 1). However, obese women were less likely to have PTB (AOR 0.65, 95% CI 0.49-0.84) (Tables 2 and 3, Figure 2). Infants of obese women had an almost fourfold increased risk of being macrosomic (AOR 3.73, 95% CI 2.33-5.98); however, they were less likely to be born with LBW. However, obesity had no effect on PTB or IUGR (Tables 2 and 3). Overweight women were at increased risk of CS delivery (AOR 1.25, 95% CI 1.03-1.5) and failure of IOL (AOR 1.69, 95% CI 1.09-2.60) when compared to normal weight women.

Underweight women had two and half fold more chances of delivering an LBW infant (AOR 2.49, 95% CI, 1.58-3.92). Univariate analysis showed that underweight women had significantly more PTBs compared to normal weight women (Table 2); however, this association did not persist after controlling for covariates (Table 3, Figure 1). Being underweight did not influence any other maternal or prenatal outcomes (Table 3, Figure 2).

## 4. Discussion

The prevalence of obesity reported in this study of 38% is far higher than that reported from other high-income European countries where the rate of obesity is between 6 and 10% [27, 28]. However, it is similar to prepregnancy obesity and overweight observed in other Middle Eastern countries [29], which may be explained partially by the differences in sociodemographic, lifestyle, and pregnancy characteristics between different ethnic groups [29]. Nevertheless, the rate of maternal prepregnancy overweight and obesity of 68% documented in this study is nearly 20% over the rate reported during 2009 from Saudi Arabia [8, 30].

Similar to our findings, earlier studies, including those from Saudi Arabia, showed that increased maternal age and parity are associated with higher risk of prepregnancy overweight and obesity [13, 30].

Our findings showed that prepregnancy obesity is associated with an increased risk of developing GDM, when compared to normal weight women. Similar results were previously reported by El-Gilany and Hammad [30], with a noticeable lower rate of GDM compared to the current cohort, which may be explained by the difference in diagnostic criteria for GDM and the higher prevalence of overweight and obesity in this study compared to the previous cohort.

The risk of developing GDM was found to increase by 4% with each additional unit of prepregnancy BMI compared to the risk of normal weight women [31]. Physiological changes of pregnancy are directed at increasing the deposition of adipose tissue in early pregnancy, which is used in late pregnancy to increase free fatty acids (FFA) through lipolysis [32, 33]. This process is accompanied by a reduced use of free glucose due to an increase in the peripheral resistance to insulin [32, 34]. In women with pre-pregnancy obesity, lipolysis and insulin resistance exceed the physiological threshold hence, are more prone to develop GDM, and their neonates are more likely to be macrosomic due to the abundance of FFA and glucose crossing the placenta and metabolized in the fetal liver [35].

Similar to our findings, other investigators confirmed the association between prepregnancy obesity and hypertensive disorders of pregnancy [36, 37]. The excess of circulating metabolically active compounds such as glucose, insulin, FFA, and leptin in obese women is likely to increase the risk of hypertensive disorders of pregnancy [38].

As shown in our study, infants of obese mothers were more likely to be macrosomic and less likely to be preterm [28].

Concurred with our findings, other reports confirmed that obese women were at increased risk of experiencing IOL [39]. They are less likely to have spontaneous labour and tend to have postterm pregnancy, as shown in previous studies [39, 40]. In addition to the increased frequency of obstetric morbidities, such as GDM and hypertensive disorders among obese women which is directly associated with the increased need of IOL, moreover, they are more likely to experience failure of IOL and an increased risk of CS delivery compared to normal weight women [41, 42]. This may be explained by the abnormal uterine contractions and dysfunctional labour noticed among obese women [43].

This study confirmed that, the odds of developing adverse maternal or perinatal outcomes were less frequent in overweight women compared to those who were obese but more frequent than normal weight ones. This observation shows a rather linear relationship between prepregnancy weight and adverse pregnancy outcomes.

Underweight women were at increased risk of delivering LBW infant, but we did not find any association between PTB and prepregnancy underweight or obesity after we controlled for covariates.

The findings of previous studies on the association between different categories of prepregnancy BMI and PTB are conflicting. Results of a systematic review and meta-analysis which included 78 studies concluded that maternal prepregnancy underweight is associated with both spontaneous and indicated PTB [16]. However, the meta-analysis in this review was associated with a high degree of heterogeneity; in addition, most of the included studies did not control for covariates of PTB, therefore reducing the certainty of the body of evidence [16]. Other investigators found an association between maternal obesity and PTB [44] and suggested an inflammatory process mediated by the acute phase C-reactive protein as being responsible for this outcome [45].

Our findings on the association between prepregnancy underweight and LBW have been reported by other investigators [2, 46], and these findings may be related to maternal malnutrition and/or anemia [47].

Some associations between prepregnancy weight and adverse outcomes, which we did not observe in this cohort, including IUGR, stillbirth, and other parameters that we did not investigate, such as early and late neonatal death. The variation in the associations between a number of outcomes and prepregnancy weight in different cohorts may be explained by different lifestyles and sociodemographic characteristics among communities and different ethnic groups.

### 4.1. Implication to Practice

Although our estimated risks for maternal and perinatal complications of prepregnancy weight are similar to the ones of other investigators, the impact of prepregnancy obesity and overweight on the Saudi pregnant women is expected to be greater due to the high prevalence of obesity and overweight compared to other communities, calling for urgent measures including the following:

1. Early screening during pregnancy, for obesity, overweight, and underweight, hence categorizing women with prepregnancy weight problems as high-risk for maternal and perinatal complications
2. Monitoring of gestational weight gain (GWG) for women with prepregnancy obesity and underweight can improve some of outcomes of pregnancy, especially if it is kept within the recommendation of the Institute of Medicine for weight gain in pregnancy [48, 49]
3. As obesity in pregnancy is a major public health problem, it is mandatory to establish national guidelines for the screening and management of obese women during pregnancy which will standardize care and improve outcomes
4. Saudi women in the reproductive age group should receive health education on the serious adverse effects of obesity on their reproductive health. Such health education can be integrated in school and university education
5. Establishment of health education programs including healthy nutrition during pregnancy and postpartum period to prevent excessive weight gain during pregnancy and postpartum.
6. Establishment of a national prevention program for obesity and its complications with evidence-based interventions at three levels; primary, secondary and tertiary levels.

### 4.2. Implication to Research

Further research should be directed to the investigation of biological and molecular effects of maternal obesity on the neonate, child, and adult future health. In addition, research should be directed towards effective interventions to reduce the burden of prepregnancy obesity and its adverse effects.

### 4.3. Strength and Limitations

This is the first study from Saudi Arabia to investigate the effect of prepregnancy weight on different maternal and perinatal outcomes in a large cohort of over 7,000 participants. The study gives an accurate and specific account of the effects of prepregnancy weight on the outcome of pregnancy in Saudi mothers. It will provide important information for health services planning of interventions to reduce prepregnancy obesity and overweight, as well as for providing high risk pregnancies of women with high BMI with specific healthcare. We are aware of the limitation of this study including the observational nature of the investigation and the lack of data on some outcomes such as the neonatal mortality.

## 5. Conclusion

The prevalence of prepregnancy overweight and obesity is very high in Saudi Arabia and has increased by 20% over the last decade. Prepregnancy obesity is associated with increased risk of developing GDM and gestational hypertension. Furthermore, it is associated with increased obstetric complications including IOL, failed IOL, and emergency CS delivery. Infants of obese mothers were at risk of macrosomia. Overweight women were at increased risk of CS delivery and failed IOL, while underweight women were at increased risk of delivering LBW infants.

## Figures and Tables

**Table 1:** Maternal characteristics of the studied population.

Characteristics	Underweight (BMI < 18.5 kg/m^2^) *N* = 157 (2.2%)	Normal (BMI < 25 kg/m^2^) *N* = 2,087 (29.7%)	Overweight (BMI = 25-29.9 kg/m^2^) *N* = 2,338 (33.3%)	Obese (BMI ≥ 30 kg/m^2^) *N* = 2,447 (34.8%)	*P* value
Age (years)	26.0 ± 4.5	27.9 ± 5.4	29.7 ± 5.8	31.5 ± 5.8	
<20	6 (3.8)	73 (3.5)	55 (2.4)	33 (1.3)	<0.01
20-29	122 (77.7)	1,272 (60.9)	1,159 (49.6)	903 (36.9)	
30-34	22 (14.0)	459 (22.0)	625 (26.7)	746 (30.5)	
>35	7 (4.5)	283 (13.6)	499 (21.3)	765 (31.3)	
Education					
Illiterate	3 (2.5)	34 (2.2)	50 (3.1)	57 (3.6)	<0.01
School (1-12 years)	76 (63.9)	801 (52.7)	844 (52.3)	959 (59.8)	
University/above	40 (33.6)	684 (45.0)	719 (44.6)	589 (36.7)	
Work					
Housewife	137 (90.1)	1,681 (83.4)	1,845 (82.8)	1,986 (86.0)	<0.01
Employee	12 (7.9)	305 (15.1)	366 (16.4)	305 (13.2)	
Students	3 (2.0)	29 (1.4)	17 (0.8)	17 (0.7)	
Married to first cousin	38 (24.1)	512 (24.5)	600 (25.7)	610 (24.9)	0.84
Passive exposure to tobacco smoke	24 (20.2)	419 (24.8)	461 (24.3)	550 (26.8)	0.15
Preexisting hypertension	0 (0.0)	11 (0.5)	23 (1.0)	56 (2.3)	<0.01
Preexisting diabetes	0 (0.0)	38 (2.6)	57 (3.6)	102 (5.7)	<0.01
Parity	1.25 ± 1.63	1.61 ± 1.68	2.3 ± 2.1	3.0 ± 2.4	
Primiparous	73 (46.5)	702 (33.6)	507 (21.7)	343 (14.0)	<0.01
Multiparous [1–4]	66 (42.0)	1,036 (49.6)	1,181 (50.5)	1,091 (44.6)	
Grand multiparous (≥5 deliveries)	18 (11.5)	349 (16.7)	649 (27.8)	1,011 (41.3)	

BMI: body mass index. Data expressed as *n* (%) or the mean ± standard deviation.

**Table 2:** The effects of prepregnancy BMI on maternal and neonatal outcomes.

	Underweight (BMI < 18.5 kg/m^2^) *N* = 157	Normal (BMI < 25 kg/m^2^) *N* = 2,087	Overweight (BMI = 25-29.9 kg/m^2^) *N* = 2,338	Obese (BMI ≥ 30 kg/m^2^) *N* = 2,447	*P* value
Gestation diabetes	14 (13.5)	251 (18.0)	334 (21.4)	593 (33.3)	< 0.01
Hypertensive events	0 (0.0)	22 (1.1)	29 (1.2)	63 (2.6)	< 0.01
Gestational age at delivery	38.37 ± 2.30	38.73 ± 1.98	38.9 ± 1.7	38.8 ± 1.9	
PTB (<34 weeks)	6 (3.8)	43 (2.1)	26 (1.1)	49 (2.0)	0.01
PTB (34-36 weeks)	10 (6.4)	146 (7.1)	130 (5.6)	124 (5.1)	
Full term (37-41 weeks)	140 (89.7)	1,879 (90.9)	2,174 (93.3)	2,256 (92.9)	
Postdate delivery (> 41 weeks)	0 (0.0)	29 (1.4)	39 (1.7)	51 (2.1)	0.11
Induction of labour	19 (12.1)	347 (16.7)	392 (16.8)	477 (19.6)	<0.01
Failed induction of labour	1 (0.06)	41 (11.8)	66 (16.8)	91 (19.0)	0.02
Mode of delivery					
Spontaneous vaginal delivery	125 (80.6)	1,619 (78.2)	1,759 (75.7)	1,642 (67.4)	<0.01
Instrumental delivery	12 (7.7)	111 (5.4)	105 (4.5)	92 (3.8)	
Elective caesarean section	7 (4.5)	102 (4.9)	148 (6.8)	305 (12.5)	
Emergency caesarean section	11 (7.1)	239 (11.9)	303 (13.0)	396 (16.3)	
Maternal admission to ICU	1 (0.6)	10 (0.5)	5 (0.2)	9 (0.4)	0.46
Apgar less than 7 at 5 minutes	3 (1.9)	27 (1.3)	24 (1.0)	32 (1.3)	0.66
Stillbirth	1 (0.6)	14 (0.7)	16 (0.7)	16 (0.7)	0.99
Birth weight (kg)					
Low birth weight (<2.5 kg)	37 (23.6)	240 (11.5)	167 (7.1)	171 (7.0)	<0.01
Macrosomia (≥4 kg)	1 (0.6)	24 (1.3)	51 (2.3)	125 (5.5)	<0.01
Intrauterine growth restriction	6 (3.8)	40 (1.9)	23 (1.0)	23 (0.9)	<0.01
NICU	5 (3.2)	73 (3.5)	65 (2.8)	98 (4.0)	0.14

BMI: body mass index; ICU: intensive care unit; NICU: neonatal intensive care unit; PTB: preterm birth. Data expressed as *n* (%) or the mean ± standard deviation.

**Table 3:** Adjusted effect of prepregnancy BMI on pregnancy outcomes.

Pregnancy outcome	OR (95% CI.)	AOR (95% CI)
GDM		
Normal (BMI < 25 kg/m^2^) (*N* = 2,037)	1	1
Underweight (BMI < 18.5 kg/m^2^) (*N* = 157)	0.71 (0.40-1.26)	0.75 (0.42-1.34)
Overweight (BMI = 25-29.9 kg/m^2^) (*N* = 2,338)	1.30 (1.08-1.56)	1.16 (0.96-1.40)
Obese (BMI ≥ 30 kg/m^2^) (*N* = 2,447)	2.49 (2.11-2.95)^∗^	2.07 (1.73-2.47)^∗^
Hypertensive events		
Normal (BMI < 25 kg/m^2^) (*N* = 2,037)	1	1
Underweight (BMI < 18.5 kg/m^2^) (*N* = 157)	0	0
Overweight (BMI = 25-29.9 kg/m^2^) (*N* = 2,338)	1.18 (0.78-1.78)	1.21 (0.69-2.15)
Obese (BMI ≥ 30 kg/m ^2^) (*N* = 2,447)	2.60 (1.81-3.72)^∗^	2.33 (1.49-3.91)^∗^
Induction of labour		
Normal (BMI < 25 kg/m ^2^) (*N* = 2,037)	1	1
Underweight (BMI < 18.5 kg/m^2^) (*N* = 157)	0.69 (0.42-1.13)	0.72 (0.44-1.18)
Overweight (BMI = 25-29.9 kg/m^2^) (*N* = 2,338)	1.01 (0.86-1.19)	1.06 (0.93-1.25)
Obese (BMI ≥ 30 kg/m^2^) (*N* = 2,447)	1.22 (1.05-1.41)^∗^	1.40 (1.19-1.65)^∗^
Emergency CS		
Normal (BMI < 25 kg/m^2^) (*N* = 2,037)	1	1
Underweight (BMI < 18.5 kg/m^2^) (*N* = 157)	0.53 (0.31-1.09)	0.55 (0.29-1.04)
Overweight (BMI = 25-29.9 kg/m^2^) (*N* = 2,338)	1.15 (0.96-1.37)	1.248 (1.03-1.50)^∗^
Obese (BMI ≥ 30 kg/m^2^) (*N* = 2,447)	1.49 (1.26-1.78)^∗^	1.67 (1.39-2.01)^∗^
Failed induction of labour		
Normal (BMI < 25 kg/m^2^) (*N* = 347)	1	1
Underweight (BMI < 18.5 kg/m^2^) (*N* = 19)	0.41 (0.05-3.16)	0.60 (0.08-4.68)
Overweight (BMI = 25-29.9 kg/m^2^) (*N* = 392)	1.51 (1.01-2.29)^∗^	1.69 (1.09-2.60)^∗^
Obese (BMI ≥ 30 kg/m^2^) (*N* = 477)	1.12 (2.62)^∗^	2.13 (1.40-3.25)^∗^
PTB		
Early PTB (<34 weeks)		
Normal (BMI < 25 kg/m^2^) (*N* = 2,037)	1	1
Underweight (BMI < 18.5 kg/m^2^) (*N* = 157)	1.88 (0.79-4.50)	1.94 (0.81-4.64)
Overweight (BMI = 25-29.9 kg/m^2^) (*N* = 2,338)	0.53 (0.33-0.87)^∗^	0.53 (0.33-0.88)^∗^
Obese (BMI ≥ 30 kg/m^2^) (*N* = 2,447)	0.97 (0.64-1.47)	0.94 (0.60-1.46)
PTB (34-36 weeks)		
Normal (BMI < 25 kg/m^2^) (*N* = 2,037)	1	1
Underweight (BMI < 18.5 kg/m^2^) (*N* = 157)	1.11 (0.65-1.92)	0.96 (0.50-1.87)
Overweight (BMI = 25-29.9 kg/m^2^) (*N* = 2,338)	0.72 (0.57-0.89)^∗^	0.73 (0.59-0.93)^∗^
Obese (BMI ≥ 30 kg/m^2^) (*N* = 2,447)	0.77 (0.62-0.95)^∗^	0.65 (0.49-0.84)^∗^
Macrosomia (≥ 4 kg)		
Normal (BMI < 25 kg/m^2^) (*N* = 2,037)	1	1
Underweight (BMI < 18.5 kg/m^2^) (*N* = 157)	0.64 (0.08-4.76)	0.76 (0.10-5.68)
Overweight (BMI = 25-29.9 kg/m^2^) (*N* = 2,338)	1.83 (1.11-2.98)^∗^	1.74 (1.05-2.90)^∗^
Obese (BMI ≥ 30 kg/m^2^) (*N* = 2,447)	4.42 (2.84-6.87)^∗^	3.73 (2.33-5.98)^∗^
LBW (<2.5 kg)		
Normal (BMI < 25 kg/m^2^) (*N* = 2,037)	1	1
Underweight (BMI < 18.5 kg/m^2^) (*N* = 157)	2.37 (1.60-3.52)^∗^	2.49 (1.58-3.92)^∗^
Overweight (BMI = 25-29.9 kg/m^2^) (*N* = 2,338)	0.59 (0.48-0.73)^∗^	0.72 (0.57- 0.91)^∗^
Obese (BMI ≥ 30 kg/m^2^) (*N* = 2,447)	0.58 (0.47-0.71)^∗^	0.63 (0.49-0.82)^∗^
IUGR		
Normal (BMI < 25 kg/m^2^) (*N* = 2,037)	1	1
Underweight (BMI < 18.5 kg/m^2^) (*N* = 157)	2.04 (0.85-4.89)	1.65 (0.66-4.16)
Overweight (BMI = 25-29.9 kg/m^2^) (*N* = 2,338)	0.51 (0.31-0.85)^∗^	0.62 (0.36-1.04)
Obese (BMI ≥ 30 kg/m^2^) (*N* = 2,447)	0.49 (0.29-0.81)^∗^	0.60 (0.35-1.04)

OR: crude odds ratio; AOR: adjusted odds ratio; CI: confidence intervals; GDM: gestational diabetes; PTB: preterm birth; LBW: low birth weight; IUGR: intrauterine growth restriction. The regression model was adjusted for maternal age, parity, and gestational age excepting for preterm birth that was adjusted for maternal age, parity, preexisting diabetes, gestational hypertension, and GDM.

## Data Availability

We confirm that any interested researcher can obtain a minimal data set after contacting HW at umlena@yahoo.com.

## Supplementary Materials

Supplementary Materials Comparison between included and excluded participants from the originally recruited cohort in the main determinants of the outcomes.
