# Supplementary material for: Maternal Prepregnancy Weight and Pregnancy Outcomes in Saudi Women: Subgroup Analysis from Riyadh Mother and Baby Cohort Study (RAHMA)
Source: Biomed Res Int. 2021 Apr 1;2021:6655942. doi: 10.1155/2021/6655942 (PMC8034996; doi:10.1155/2021/6655942)
Supplement: Supplementary Materials — Comparison between included and excluded participants from the originally recruited cohort in the main determinants of the outcomes. [file 6655942.f1.docx]

| Characteristic | Included  N=7029 | Excluded  N=7539 |
| --- | --- | --- |
| Age | 29.81±5.9 | 30.0±5.9 |
| Parity | 2.3±2.2 | 2.3±2.2 |
| Gestational Age | 38.8±2.1 | 38.4±2.5 |
| BMI at term | 31.8±5.8 | 31.7±5.8 |
| Birth weight | 3.1±0.5 | 3.1±0.6 |
